# The ISCON-trial protocol: laparoscopic ischemic conditioning prior to esophagectomy in patients with esophageal cancer and arterial calcifications

**DOI:** 10.1186/s12885-022-09231-x

**Published:** 2022-02-05

**Authors:** A. van der Veen, L. M. Schiffmann, E. M. de Groot, I. Bartella, P. A. de Jong, A. S. Borggreve, L. A. A. Brosens, D. Pinto Dos Santos, H. Fuchs, J. P. Ruurda, C. J. Bruns, R. van Hillegersberg, W. Schröder

**Affiliations:** 1 Department of Surgery, University Medical Center Utrecht, POBOX 85500, 3508 GA Utrecht, The Netherlands; 2 Department of General, Visceral, Cancer and Transplant Surgery, Faculty of Medicine, University of Cologne, Cologne, Germany; 3 Department of Radiology, Division of Imaging and Oncology, University Medical Center Utrecht and Utrecht University, Utrecht, The Netherlands; 4 Department of Pathology, University Medical Center Utrecht, Utrecht University, Utrecht, The Netherlands; 5 Institute of Diagnostic and Interventional Radiology, University of Cologne, Medical Faculty and University Hospital Cologne, Cologne, Germany

**Keywords:** Esophagectomy, Ischemic preconditioning, ISCON

## Abstract

**Background:**

Anastomotic leakage is the most important surgical complication following esophagectomy. A major cause of leakage is ischemia of the gastric tube that is used for reconstruction of the gastrointestinal tract. Generalized cardiovascular disease, expressed by calcifications of the aorta and celiac axis stenosis on a pre-operative CT scan, is associated with an increased risk of anastomotic leakage. Laparoscopic ischemic conditioning (ISCON) aims to redistribute blood flow and increase perfusion at the anastomotic site by occluding the left gastric, left gastroepiploic and short gastric arteries prior to esophagectomy. This study aims to assess the safety and feasibility of laparoscopic ISCON in selected patients with esophageal cancer and concomitant arterial calcifications.

**Methods:**

In this prospective single-arm safety and feasibility trial based upon the IDEAL recommendations for surgical innovation, a total of 20 patients will be included recruited in 2 European high-volume centers for esophageal cancer surgery. Patients with resectable esophageal carcinoma (cT1-4a, N0–3, M0) with “major calcifications” of the thoracic aorta accordingly to the Uniform Calcification Score (UCS) or a stenosis of the celiac axis accordingly to the modified North American Symptomatic Carotid Endarterectomy Trial (NASCET) score on preoperative CT scan, who are planned to undergo esophagectomy are eligible for inclusion. The primary outcome variables are complications grade 2 and higher (Clavien-Dindo classification) occurring during or after laparoscopic ISCON and before esophagectomy. Secondary outcomes include intra- and postoperative complications of esophagectomy and the induction of angiogenesis by biomarkers of microcirculation and redistribution of blood flow by measurement of indocyanine green (ICG) fluorescence angiography.

**Discussion:**

We hypothesize that in selected patients with impaired vascularization of the gastric tube, laparoscopic ISCON is feasible and can be safely performed 12–18 days prior to esophagectomy. Depending on the results, a randomized controlled trial will be needed to investigate whether ISCON leads to a lower percentage and less severe course of anastomotic leakage in selected patients.

**Trial registration:**

Clinicaltrials.gov, NCT03896399. Registered 4 January 2019.

**Supplementary Information:**

The online version contains supplementary material available at 10.1186/s12885-022-09231-x.

## Background

Transthoracic esophagectomy with 2-field lymphadenectomy is the standard of surgical care for patients with esophageal cancer [1]. The reconstruction of choice is a gastric tube with intrathoracic (Ivor-Lewis) or cervical esophagogastrostomy (McKeown). This gastric tube is perfused only by the right gastroepiploic artery, as all other gastric arteries are ligated during gastric mobilization. This is associated with severe change of microcirculation in the gastric tube, reducing gastric perfusion up to 50% [2–5]. Reduced blood flow may lead to impaired healing of the anastomosis and could result in anastomotic leakage. Anastomotic leakage occurs in 15–30% of patients after esophagectomy [6]. It is considered the most important complication after esophagectomy, increasing postoperative morbidity and mortality. Anastomotic leakage has a multifactorial etiology. Some risk factors have been identified, such as severe comorbidity, diabetes mellitus, smoking status, radiation field and cervical anastomosis [7, 8].

Another important risk factor is the vascular status which can be inferred from calcification in the thoracic aorta, defined by the uniform calcification score (UCS). The UCS is calculated on diagnostic CT scans by scoring the presence of arterial calcification in the thoracic aorta based on a visual grading system. In addition, the presence of a local stenosis of the celiac trunk is also associated with an increased risk of anastomotic leakage [9]. This stenosis is defined by the modified North American Symptomatic Carotid Endarterectomy Trial score (modified NASCET score). Higher percentages of anastomotic leakage (33–37%) were observed in patients with calcifications compared to patients without calcifications, who had lower incidences of leakage (9–19%) [10–12].

The higher prevalence of anastomotic leakage in these patients are hypothesized to be the result of a reduced micro or macro perfusion of the gastric tube [2–4, 13]. Anastomotic leakage percentages might be reduced by ischemic conditioning (ISCON) of the gastric tube. ISCON aims to increase perfusion at the anastomotic site by redistribution of the gastric blood flow [14]. This is achieved by occluding all of the gastric arteries except the right gastric and right gastroepiploic artery during a separate intervention, days or weeks prior to esophagectomy.

To date, several studies reported ISCON to be safe in esophageal surgery and its possible efficacy in decreasing anastomotic leakage [15, 16]. However, all studies were retrospective and performed in unselected patients. Therefore, the current prospective safety and feasibility trial aims to investigate the feasibility and safety of performing laparoscopic ISCON for esophageal cancer in patients at high-risk for anastomotic leakage, as based upon their vascular status on pre-operative CT scans (defined by the UCS of the thoracic aorta and modified NASCET score of the celiac axis). The hypothesis of this study is that in these selected patients with an increased risk of vascular impairment of the gastric tube, laparoscopic ISCON is feasible and can be safely performed prior to esophagectomy.

## Methods/design

### Design

This study is designed as a prospective single-arm safety and feasibility trial performed at the University Medical Center Utrecht and the University Hospital of Cologne.

### Ethical consideration

The study protocol was approved by the Medical Ethical Committees of the University Medical Center Utrecht (reference number NL67819.041.18) and the University of Cologne (reference number 18–299). The trial was prospectively registered at clinicaltrials.gov.

### Patient population

All patients with a resectable esophageal carcinoma (cT1-4aN0-3 M0) scheduled for an esophagectomy are eligible for screening for inclusion in the study. Accordingly to policies in the Netherlands and Germany, included patients undergo neoadjuvant chemo(radio) therapy followed by laparoscopic ISCON and subsequent esophagectomy. An exception will be made for patients with early esophageal cancer (cT1-2N0M0) and patients who are not fit enough for neoadjuvant treatment, they will bypass neoadjuvant treatment and undergo primary ISCON followed by esophagectomy. Detailed inclusion and exclusion criteria are listed below.

Inclusion criteria:

- Histologically proven adenocarcinoma or squamous cell carcinoma of the esophagus or gastroesophageal junction
- Planned to undergo transthoracic esophagectomy or transhiatal esophagectomy
- Preoperative CT-scan
- Arterial calcifications: “major calcifications” of the thoracic aorta according to the (UCS) or a stenosis of the celiac axis according to the modified NASCET score
- ASA classification I-III
- European Clinical Oncology Group (ECOG) performance status of 0–2
- Age > 17
- Written informed consent

Exclusion criteria:

- Not able to undergo study treatment
- Presence of metastatic disease

### UCS and NASCET score

Preoperative staging examinations of all patients are routinely performed on CT scanners with 64 detector rows or more. A slice thickness of maximum 3.0 mm is used. Two clinicians (of whom at least one is a radiologist) will independently score the calcifications on the preoperative CT scans of the thoracic aorta (UCS score) and the celiac axis (modified NASCET score). Any disagreements will be solved based on discussion. The UCS will be used in order to consistently score CT images on arterial calcification at the thoracic aorta (heart – celiac axis). Scores of 0, 1 or 2 will be assigned, corresponding with absent, minor or major calcifications, respectively (see Table 1). Stenosis of the celiac axis will be evaluated by using multiplane reconstructions. Accordingly to the NASCET score, the diameters of the normal (a) and narrowest (b) lumen of the celiac axis will be measured. The percent stenosis (s) will be calculated using the following formula: s = (a-b)/a × 100.

**Table 1:**
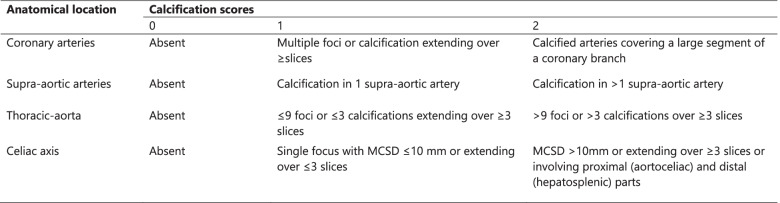
Uniform Calcification Score: Definitions used to visually grade arterial calcification on preoperative CT images. MCSD: maximum cross-sectional diameter

*these anatomical locations are secondary outcome measures

### Laparoscopic ISCON

The first operation aims to partially devascularize the stomach by laparoscopic clipping of the left gastric artery, reached through the hepatogastric ligament at the lesser curvature. Furthermore, transection (with the harmonic scalpel or comparable instrument) of the short gastric vessels including the left gastroepiploic artery is performed. This operation will be kept as minimalistic as possible. Vascularization of the right gastric artery and the right gastroepiploic artery along the greater curvature will remain preserved. No lymph node dissection or gastric tube formation will be performed.

### Postoperative management ISCON

Patients are allowed to drink liquids, soup and supplemental nutrition drinks (high energy, high protein oral nutritional supplements) on postoperative day 0. All patients will have a form of liquid or solid enteral nutrition between ISCON and esophagectomy. Patients will be eligible for discharge on postoperative day 3, depending on the clinical course. A dietician will be consulted to ensure that the patient is optimized in terms of nutrition between ISCON and esophagectomy. All patients will receive a mandatory outpatient clinic standard follow-up appointment on postoperative day 6–8, unless they are still admitted at the hospital. Patients will be re-admitted 0–1 day before esophagectomy.

### Esophagectomy

Esophagectomy will be performed after an interval of 12–18 days after ISCON. If a gastroparesis is suspected, a nasogastric tube will be placed before anaesthesia to avoid aspiration. Esophagectomy will consist of a transthoracic esophagectomy with intrathoracic or cervical anastomosis or a transhiatal esophagectomy with cervical anastomosis. In the University Hospital of Cologne, an intrathoracic anastomosis will be created in all patients except for those with a tumor localized in the cervical compartment. In the University Medical Center Utrecht, a cervical anastomosis will be created for proximal and mid esophageal tumors while an intrathoracic anastomosis will be created for distal esophageal tumors. Alternatively, instead of a transthoracic esophagectomy, a transhiatal esophagectomy can be performed in patients with increased comorbidity.

The esophagectomy includes laparoscopic gastric mobilization, abdominal lymph node dissection, intrathoracic lymph node dissection (for transthoracic esophagectomies), esophagectomy and intrathoracic or cervical anastomosis. The abdominal phase will be performed as a minimally invasive procedure, the thoracic procedure can be performed by an open or a (robot assisted) thoracoscopic approach.

If, for any reason, it is not possible to perform the second operation within 12–18 days, it will be attempted to perform the second operation as soon as possible (i.e. within 30 days) based on the discretion of the surgeon who performed the first operation.

### Translational program

To assess the effect of laparoscopic ISCON on macro- and microcirculation, a translational program is included in the study. This program consists of two parts: measurements of macro- and microcirculation. This will be investigated by means of blood samples (cytokine profile), biopsies (vascularity) and ICG fluorescence angiography. Blood samples will be collected before ISCON and esophagectomy and will be screened on biomarkers. The presence and the level of biomarkers will be compared in the blood samples before and after ISCON to detect potential changes. Biopsies will be taken via gastroscopy preoperatively to ISCON as well as esophagectomy, either within 24 h before surgery or immediately after anesthesia. Three biopsies will be taken from the gastric fundus since the anastomosis will likely be located somewhere in the fundus. In order to identify the fundus, the endoscopy will be performed right after the laparoscopic camera is inserted so that the table surgeon is able to point out the fundus. Finally, if a stapler is used for performance of the anastomosis, the gastric anastomotic donut will be collected and if the anastomosis is hand-sewn, the tip of the gastric tube will be collected to for further pathological analysis and to detect morphological changes of the microvasculature. ICG will be performed during ISCON, before and after the occlusion of the gastric arteries and during esophagectomy, before the creation of the gastric conduit and optionally before the creation of the intrathoracic anastomosis. The ICG procedure is standardized and included in the protocol as described in Supplementary 1. The goal is to quantify the effect of laparoscopic ISCON on gastric perfusion which is described in Supplementary 2. During ICG, the camera keeps the gastric fundus in view. If ICG is also performed for the anastomosis, the camera keeps the gastric conduit in view. The different time points of the translational program are summarized in Table 2.

**Table 2:** Summary of time points of the translational program. h = hour

	ICG fluorescence	Biomarkers
< 24 h to start of ISCON		Biopsies of anastomotic site + peripheral blood (10 ml EDTA)
ISCON: before occlusion of arteries	X	
ISCON: 10 min after occlusion of arteries	X	
< 24 h to start of esophagectomy		Biopsies of anastomotic site + peripheral blood (10 ml EDTA)
Esophagectomy: before formation of gastric tube	X	
Optional: esophagectomy: before formation of intrathoracic anastomosis	(X)	
Esophagectomy: after completion of anastomosis		Gastric anastomotic donut or tip of gastric tube

### Primary outcome

Complications are defined according to the Esophageal Complications Consensus Group (ECCG) and graded according to the Clavien Dindo Classification [1, 17]. The primary outcome measure is the percentage of complications grade ≥ 2 occurring during or after ISCON and before esophagectomy.

### Secondary outcomes

The main secondary outcome measures include all grade 1 complications occurring during or after ISCON and before esophagectomy according to the Clavien Dindo Classification. Intraoperative outcomes will be scored during both surgeries including the presence of adhesions, intraoperative complications and the vascularisation of the stomach (based on the color of the tissue). Furthermore, for ISCON, the duration of the procedure, blood loss, oral intake, weight and day of discharge will be collected. Lastly, 30 day mortality, anastomotic leakage of any grade and all other postoperative complications grade ≥ 3b will be collected after esophagectomy.

### Translational outcomes

Secondary outcomes regarding the translational program are induction of angiogenesis by biomarkers of microcirculation, redistribution of blood flow by measurement of indocyanine green (ICG) fluorescence angiography. Serum levels of several proangiogenic cytokines (VEGF, IL-8, IL-6, TNF-α and Ang-2) will be determined in peripheral blood samples by ELISA [18]. The obtained biopsies and gastric donut samples/tip of the gastric conduit will be collected from the endoscopy unit or the operation theatre and will be fixed in formaldehyde. Paraffin embedded sections (10 μm) will be stained by immunohistochemistry against CD31 (vessel density) or smooth-muscle-actin positive pericytes to detect morphological changes of the microvasculature. The strength of the ICG will be scored on videos that are recorded during the operation in 2 fashions, subjectively and objectively.

### Data collection

All data will be collected and stored in an electronic Clinical Research Form application called OpenClinica. The coordinating investigators will oversee the overall data collection process. OpenClinica generates a subject number for each patient and securely stores all entered research data in a pseudonymized fashion. A code file that links subjects numbers to individual patients will be securely stored in each center, and will only be accessible to the local study coordinators. Collected baseline data (age, gender, body mass index, comorbidities) and treatment details (neoadjuvant therapy, surgical techniques, postoperative complications, mortality) will be prospectively entered in a case report form with built-in validation checks. Painscores according to numeric rating scale 0-10 will be collected at postoperative day 1-3 after ISCON. In addition, radiological findings at the coronary arteries, supra-aortic arteries and celiac axis (UCS) will be scored. Patient-reported outcome measures (PROMs) will be collected prior to both surgeries by asking patients to complete the questionnaires (EORTC QLQ-C30 and QLQ-OG25).

### Sample size

This is a prospective single arm safety and feasibility trial, classified as a “stage 2a Development” study according to the IDEAL recommendations for surgical innovation [19]. Based upon these recommendations, a total of 20 subjects will be included and no formal sample size calculations are performed (10 patients per centre).

### Statistical analyses

Statistical analyses in this study are primarily based on descriptive means. Categorical data will be summarized as frequencies. Normally distributed continuous data will be summarized as means with corresponding standard deviations. Non-normally distributed continuous data will be summarized as medians with corresponding interquartile ranges. Repeated outcomes at various time points (such as translational outcomes and PROMS) will be presented descriptively and tested as paired measurements analysis will be performed with SPSS software (IBM).

### Data safety monitoring board & monitoring

This study is classified as a medium risk study. Monitoring will be performed in both centers by an external independent Contract Research Organization according to the Study Monitoring Plan. Based upon advice of the Data Safety Monitoring Board (DSMB) of the UMC Utrecht, a DSMB will not be assigned. Instead of a DSMB, an independent Medical Safety Officer -a Professor in Gastroenterology- was assigned to perform ongoing safety surveillance together with the researchers. After the first 5 patients underwent esophagectomy, all serious adverse events (SAEs) will be evaluated with the Medical Safety Officer. After the first 10 patients underwent both surgeries, an interim analysis will be performed which is described in detail below. All SAEs will be reported by the sponsor to the ethical committee and to the Medical Safety Officer.

### Interim analysis

An interim analysis will be performed after hospital discharge (after esophagectomy) of the first 10 patients included in the study. The stopping rules are the occurrence of one of the following:

- > 40% postoperative complication of gastric perforation requiring re-intervention, during or after laparoscopic ISCON and before esophagectomy.
- > 40% patients having an aspiration pneumonia after laparoscopic ISCON, resulting in a prolonged hospital stay.
- > 40% patients not able to undergo the planned esophagectomy within 30 days after laparoscopic ISCON, due to complications specifically attributed to laparoscopic ISCON.

## Discussion

Anastomotic leakage is the predominant surgical complication after esophagectomy [1]. The cause of an anastomotic leakage is multifactorial [7, 8]. One of these factors includes hypovascularization of the gastric conduit which opposes healing of the anastomosis and consequently contributes to leakages. In retrospective analyses of both participating centres of the current study, major calcifications of the aorta and celiac axis have been shown to be an independent risk factor for anastomotic leakage [20, 21]. The preoperative identification of patients at risk for anastomotic leakage allows for personalized treatment programs. The ISCON trial is a safety and feasibility study aiming to stimulate the vascularisation of the gastric conduit prior to the esophagectomy in selected patients. Patients at high risk for anastomotic leakage are selected via calcification scores on preoperative CT-scans.

### Efficacy ISCON

Several studies have investigated the efficacy of ISCON. A recent meta-analyses published in 2020 showed that ISCON seems to reduce the incidence and severity of anastomotic leakage [22]. Yet ISCON failed to demonstrate a significant reduction of leakage precentages in other meta-analyses and systematic reviews of clinical studies [16, 23, 24]. One explanation for this controversy could be the fact that multiple factors contribute to the development of anastomotic leakage ensuing that ISCON alone might not have enough impact to significantly decrease anastomotic leakage numbers. However, this discrepancy could also be explained by the heterogeneity and retrospective nature of the studies. The heterogeneity is caused by several factors including the selection of patients, the time interval between ISCON and esophagectomy, which arteries are occluded and the technique that is used for ISCON (radiological versus laparoscopic). These factors could have influenced the efficacy and are discussed separately in the consecutive paragraphs.

### Selection of patients

The majority of the studies reporting on ISCON did not select patients [24]. In the current study, the UCS for major calcifications of the thoracic aorta was used as an indicator of poor generalized cardia vascular status. The location of thoracic aorta was internally and externally validated as a predictor for anastomotic leakage after esophagectomy in 3 studies [13, 20, 25].

### Interval

Another discussion point is the interval between ISCON and esophagectomy which has been widely discussed in the literature [24]. On the one hand, the interval should be long enough to redistribute the blood flow of the stomach. On the other hand, the interval should be short enough to avoid hindering the esophagectomy due to potential adhesions or causing a delay in the treatment. In the literature, intervals range between 4 and over a 100 days. Increasing evidence is available arguing for an interval of 2 weeks. Animal studies have demonstrated that immediately after ISCON, the gastric perfusion drops to 20–30%. After 1 week, the gastric perfusion around 60% and 2 weeks after ISCON, the gastric perfusion is over 90% [26, 27]. In addition, a recent meta-analysis compared the studies with an interval of > 2 weeks versus < 2 weeks and showed a trend towards lower leakage percentages for > 2 weeks, whereas no reduction in leakages was seen for < 2 weeks [22]. In addition, an interval of 2 weeks is likely to be short enough to prevent the development of adhesions. In order to minimalize adhesions, ISCON procedure is kept as lean as possible and no further preparations for the esophagectomy will be performed during the first operation. The presence of adhesions during esophagectomy will be scored accordingly to the Peritoneal adhesion index [28].To take into account for logistics, as scheduling surgeries and weekends, an interval of 12 till 18 days between ISCON and esophagectomy is used in the current trial.

### Radiological versus surgical ISCON

In the current study, ISCON is performed by occluding the left gastric, left gastroepiploic and short gastric arteries in an attempt to achieve a maximum ischemic effect. In contrast, some of the retrospective studies occluded only the left gastric artery [24]. Furthermore, ISCON could be performed radiologically as well as surgically. Superiority of one technique over the other has not yet be demonstrated. In the current study, ISCON will be performed laparoscopically since our study team already has experience with this procedure. In addition, a potential benefit of surgical ISCON is the precise and certain occlusion of the target arteries. Radiologically, it could be more difficult to selectively occlude the short gastric arteries. Instead of the short gastrics, the splenic artery could be partly embolized with a risk of splenic ischemia [29]. Furthermore, surgical ISCON allows for a translational arm with ICG measurements. Advantages of radiological ISCON are that it can be performed under local anesthesia and it does not cause adhesions.

### Safety ISCON

A meta-analysis on ISCON was published in 2017 including 11 retrospective studies and a combined total of 1152 patients. None of the included studies reported any major complications associated with the ISCON procedure itself [16]. This indicates that this procedure can be safely performed in unselected patients. Furthermore, since major calcifications of the thoracic aorta according to the UCS score have a relatively high prevalence (30%), we postulated that this meta-analysis likely also include a proportion of patients with major aortic calcification, and that ISCON could thus also be safe in these selected patients [13]. None of the retrospective studies reported perforation due to ischemia after ISCON. Hence, we will remain vigilant for this complication, but deem the chance of it occurring slim. Based upon prior clinical experience in the University Hospital of Cologne, we expect gastroparesis to occur in up to 25% of patients. Since patients with gastroparesis could be at an increased risk of acquiring an aspiration pneumonia, patients’ intake and complaints as nausea and vomiting will be closely monitored and gastroparesis will be treated by emptying the stomach via nasogastric tube placement. None of the retrospective studies report on gastroparesis and nutritional intake after ISCON. This could be explained due to the retrospective data collection. In the current study we use a clear definition of gastroparesis and will prospectively collect nutritional intake data which strengthen the study. Importantly, as mentioned, the meta-analysis included only retrospective studies and had a high degree of heterogeneity with regards to selection of patients, the time interval between ISCON, which arteries are occluded and the technique that is used for ISCON (radiological versus laparoscopic) [22]. The safety and feasibility of ISCON in selected patients have not yet been demonstrated in a prospective trial.

## Conclusion

In summary, the ISCON trial is a single-arm prospective study investigating the safety and feasibility of laparoscopic ISCON 12–18 days prior esophagectomy in a highly selected group of patients at risk to develop an anastomotic leakage. In addition, a translational program is set up to assess the postulated effect of laparoscopic ISCON. The ISCON trial is unique with respect to its prospective study design and the careful selection of eligible patients by means of atrial calcifications. If the hypothesis is confirmed that ISCON is safe and feasible, a randomized controlled, multicentre trial will be set up to determine whether esophagectomy preceded by ISCON is superior over esophagectomy without ISCON in terms of anastomotic leakage.

## Supplementary Information

**Additional file 1.**

**Additional file 2.**

## Data Availability

The dataset of this study will be available from the corresponding author on reasonable request after completion.

## Abbreviations

CT: Computed tomography
DSMB: Data Safety Monitoring Board
EOCG: European Clinical Oncology Group
FDG-PET: [18F] Fluorodeoxyglucose positron emission tomography
GEJ: Gastro-esophageal junction
ICG: Indocyanine green
ISCON: Ischemic conditioning
NASCET: North American Symptomatic Carotid Endarterectomy Trial
METC: Medical research ethics committee (MREC); in Dutch: medisch ethische toetsing commissie (METC)
CDC: Clavien-Dindo Classification of surgical complications
(S)AE: (Serious) Adverse Event
UCS: Uniform calcification score
