# Supplementary material for: The ISCON-trial protocol: laparoscopic ischemic conditioning prior to esophagectomy in patients with esophageal cancer and arterial calcifications
Source: BMC Cancer. 2022 Feb 5;22:144. doi: 10.1186/s12885-022-09231-x (PMC8817569; doi:10.1186/s12885-022-09231-x)
Supplement: Supplementary file 1 — Additional file 1. [file 12885_2022_9231_MOESM1_ESM.docx]

**Supplementary 1**

**Anesthesiology:**

- Check for contra-indications: allergy for Jodium/ICG, hyperthyroidism or thyroid adenoma.
- Start of the operation: Verdye ICG vial contains 50mg of injection powder. This is dissolved by adding 20ml of sterile water (=2,5mg/ml). 2 syringes are prepared, each with 3ml solution (containing 7.5mg ICG). The syringes and vial are kept in a dark space.
- During an ICG angiography: the mean arterial pressure is kept at >70mm Hg and is kept at a constant level. ICG 3ml (7.5mg) rapid bolus is given over a peripheral venous catheter and flushed with 10ml NaCl

**Surgery, laparoscopic ischemic conditioning:**

ICG angiography 1: Laparoscopy with ischemic conditioning, ICG **prior to** occlusion of arteries

- Video of the operation is being recorded
- Position the camera activate near-infrared filter and set-up gain/background light according to the figure 1.
- Surgeon asks anesthesiologists to inject ICG (after camera has been properly positioned).
- Camera does not move for 90 seconds (even the slightest movement disrupts measuring ICG influx speed)
- Camera is free to move: close-up inspection of entire stomach (to prevent misleading non-existent demarcations due to a difference in depth of the camera to the relevant structures, giving a weaker signal)

ICG angiography 2: Laparoscopy with ischemic conditioning, ICG **10 minutes after** occlusion of arteries

This ICG angiography is performed in similar fashion as the first ICG angiographies. The camera is set-up to visualize the exact same anatomic structures and with the exact same gain/background light.


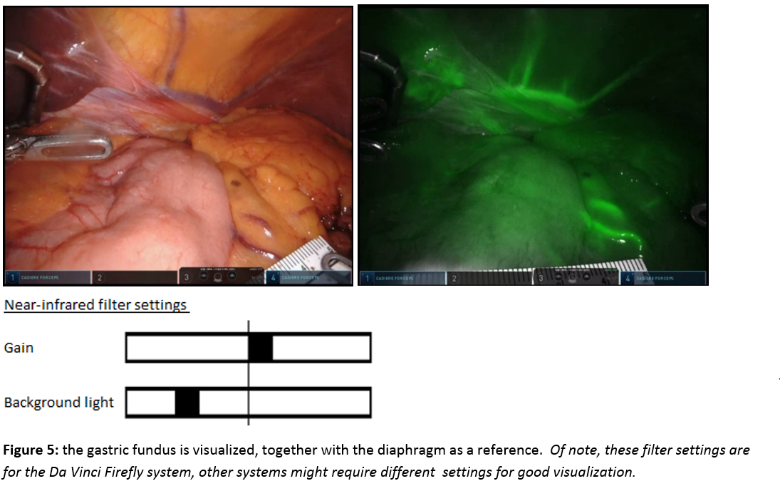


**Figure 1**

**Surgery, esophagectomy:**

ICG angiography 3: esophagectomy, ICG before formation of the gastric tube

This ICG angiography is performed in similar fashion as the first 2 ICG angiographies. An attempt is made to set-up the camera to visualize the exact same anatomic structures and with the exact same gain/background light as during the first 2 ICG angiographies.

Optional: ICG angiography 4: esophagectomy, ICG before formation of intrathoracic anastomosis

- Video of the operation is being recorded
- Position the camera, activate near-infrared filter and set-up gain/background light according to figure 2.
- Surgeon asks anesthesiologists to inject ICG (after camera has been properly positioned).
- Camera does not move for 90 seconds (even the slightest movement disrupts measuring ICG influx speed)
- Camera is free to move: close-up inspection of gastric tube up to the tip (to prevent misleading non-existent demarcations due to a difference in depth of the camera to the relevant structures, giving a weaker signal)


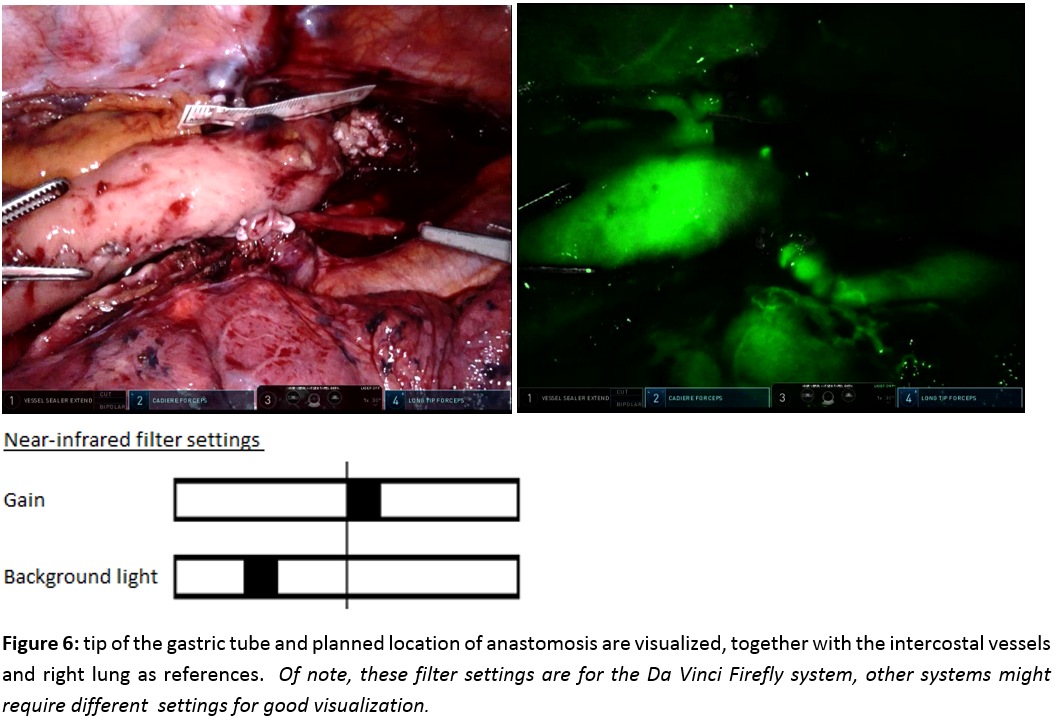


**Figure 2**
