# Supplementary material for: The ISCON-trial protocol: laparoscopic ischemic conditioning prior to esophagectomy in patients with esophageal cancer and arterial calcifications
Source: BMC Cancer. 2022 Feb 5;22:144. doi: 10.1186/s12885-022-09231-x (PMC8817569; doi:10.1186/s12885-022-09231-x)
Supplement: Supplementary file 2 — Additional file 2. [file 12885_2022_9231_MOESM2_ESM.docx]

**Supplementary 2: ISCON ICG – postoperative quantification**

The camera is positioned to visualize the area of interest (i.e. gastric fundus in abdomen measurement and proximal part of the gastric conduit where the surgeon intents to create the anastomosis in the thoracic measurement). A challenge of standardizing fluorescence measurements are factors influencing intraoperative perfusion such as differences in patient physiology, co-morbidity and intraoperative hypotension. Therefore the patient itself is used as a reference for his own physiology by visualizing controls (1 control in the abdominal measurement: the diaphragm, 2 controls in the thoracic measurement: the lung below the gastric tube and the thoracic wall above the gastric tube ). Next, ICG is administrated in a peripheral venous line (figure 1A). The control measurements should show green fluorescence first, followed by the gastric tube, indicating good perfusion of the gastric tube (as can be seen in figure 1A-E). If only the controls show green fluorescence, but the gastric tube does not, this would indicate poor perfusion of the gastric tube.


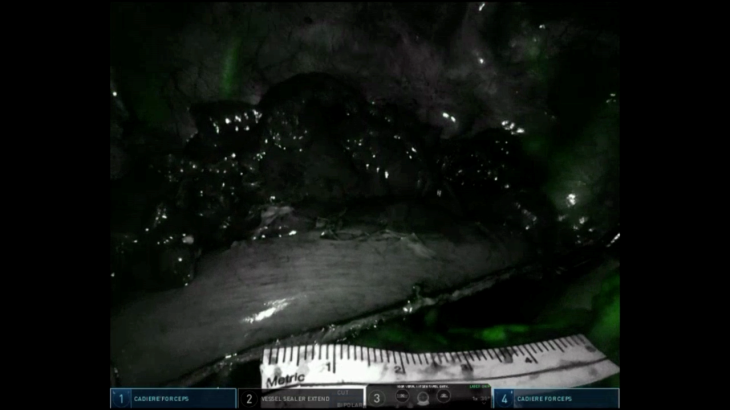


**Figure 1A.** 34 seconds after ICG injection. The grey gastric tube is centrally in the screen. The control measurement of the lung (partially blocked by the ruler) first shows green fluorescence, the thoracic wall is not yet strongly green fluorescent.


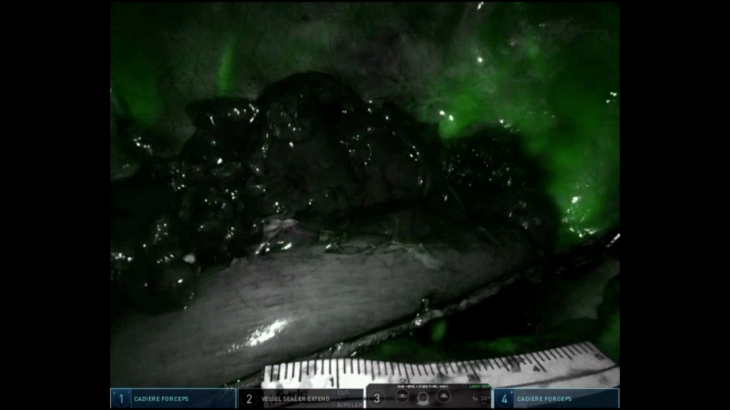


**Figure 1B.** 38 seconds after ICG injection. The control measurement of the thoracic wall also shows green fluorescence.


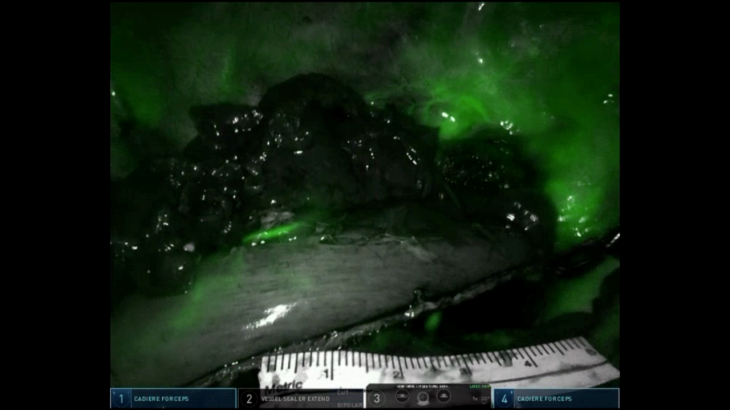


**Figure 1C.** 42 seconds after ICG injection. The more distal part of the gastric tube (left in the picture) starts to show green fluorescence.


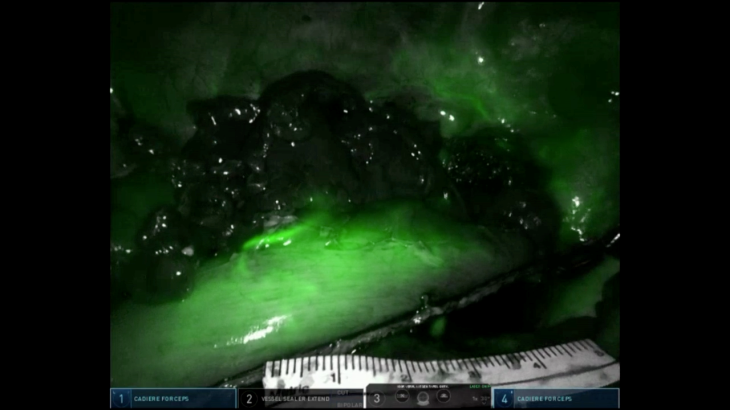


**Figure 1D.** 50 seconds after ICG injection. Both the distal and proximal part of the gastric tube show green fluorescence.


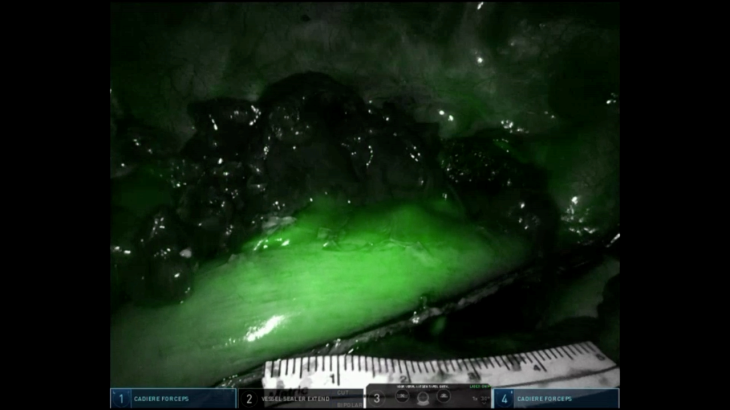


**Figure 1E.** 80 seconds after ICG injection. Green fluorescence in most proximal part of the gastric tube (right part of screen) is slightly improved compared to the image at 50 seconds.

To quantify the fluorescence, pre-defined regions of the gastric tube are selected (figure 2).

The intensity of the green fluorescence is stronger in the central vision of the camera than in the periphery. Therefore, the camera was not moved during the entire measurement. As mentioned, a challenge of standardizing fluorescence measurements are factors influencing intraoperative perfusion. To correct for the above 2 challenges, the selected regions of interest use themselves as a reference for measuring the intensity of the fluorescence. The intensity of the fluorescence (the greenness of the pixels) is measured in arbitrary units at each selected region of interest. The maximum arbitrary units is set at 100%. Next, graphs are plotted with the intensity on the Y-axis and the time on the X-axis (Figure 3). The time until reaching the maximum intensity will be calculated as the mean slope to maximum intensity (the “steepness of the graph”). In case the maximum intensity of 100% is reached quickly, the mean slope to maximum intensity is large, indicating good perfusion. If the maximum intensity of 100% is reached slowly, the mean slope to maximum intensity is small, indicating poor perfusion.


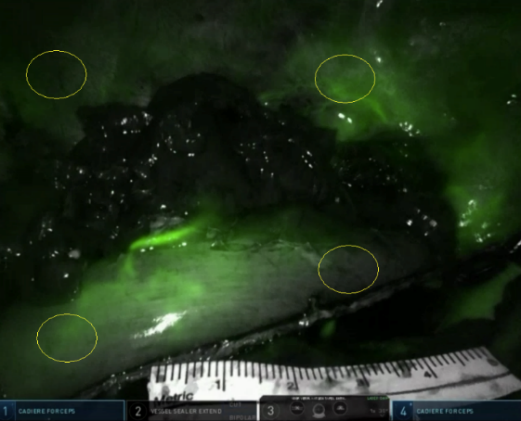


**Figure 2.** Pre-defined regions of the gastric tube are selected to quantify the fluorescence. In this case: the proximal gastric tube at 1 cm from the proximal tip and the distal gastric tube at 6 cm from the proximal tip. In addition, 2 control measurements at the thoracic wall were selected. The lung (below the gastric tube, directly above the ruler), though not used as a control in this example, is also a good site to use as a control.


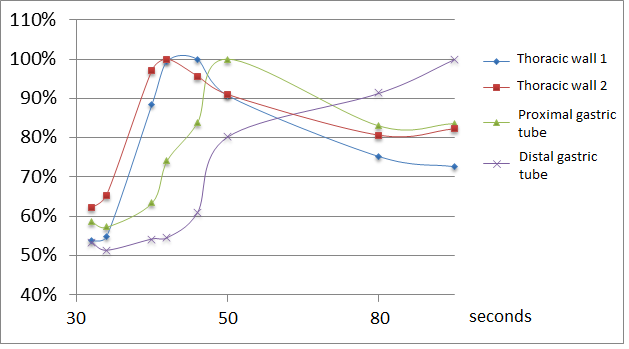


**Figure 3.** Intensity of fluorescence in % on the y-axis, time after injection of ICG in seconds on the x-axis. The graph shows how the control measurements (in this case the intrathoracic wall) show a quick rise in fluorescence (high mean slope to maximum intensity), followed by the proximal gastric tube and lastly the distal gastric tube (lower mean slope to maximum intensity). This is in accordance to normal physiology, as the gastric tube is perfused from distally by the right gastroepiploic artery.
